# Motion-based video monitoring for early detection of livestock diseases: The case of African swine fever

**DOI:** 10.1371/journal.pone.0183793

**Published:** 2017-09-06

**Authors:** Eduardo Fernández-Carrión, Marta Martínez-Avilés, Benjamin Ivorra, Beatriz Martínez-López, Ángel Manuel Ramos, José Manuel Sánchez-Vizcaíno

**Affiliations:** 1 VISAVET Center and Animal Health Department, Veterinary School, Universidad Complutense de Madrid, Madrid, Spain; 2 MOMAT Research group, IMI-Institute and Applied Mathematics Department, Universidad Complutense de Madrid, Madrid, Spain; 3 CADMS Center for Animal Disease Modeling and Surveillance, School of Veterinary Medicine, UC Davis, Davis, California, United States of America; The University of Melbourne, AUSTRALIA

## Abstract

Early detection of infectious diseases can substantially reduce the health and economic impacts on livestock production. Here we describe a system for monitoring animal activity based on video and data processing techniques, in order to detect slowdown and weakening due to infection with African swine fever (ASF), one of the most significant threats to the pig industry. The system classifies and quantifies motion-based animal behaviour and daily activity in video sequences, allowing automated and non-intrusive surveillance in real-time. The aim of this system is to evaluate significant changes in animals’ motion after being experimentally infected with ASF virus. Indeed, pig mobility declined progressively and fell significantly below pre-infection levels starting at four days after infection at a confidence level of 95%. Furthermore, daily motion decreased in infected animals by approximately 10% before the detection of the disease by clinical signs. These results show the promise of video processing techniques for real-time early detection of livestock infectious diseases.

## Introduction

Late detection of emergency diseases promotes disease spread and increases the risk of epidemic, and it may cause significant economic losses in affected countries [1], as demonstrated by the last epidemics of foot-and-mouth disease [2, 3], classical swine fever [4, 5] or the current epidemic of African swine fever (ASF) [6]. Classical strategies to detect diseases on the farm include active and passive surveillance as well as sentinel surveillance, which focuses on farms at higher risk of incursion [7].

The effectiveness of passive surveillance relies on the ability to detect disease based on the observation of clinical signs. This can pose a severe obstacle to early detection, particularly if death is the most visible clinical sign as is the case with foot-and-mouth disease, classical swine fever or ASF. On the other hand, farms may apply active surveillance approaches to gain information about disease prevalence on the farm under a sampling protocol based on appropriate laboratory tests, for example the qPCR quantitative polymerase chain reaction (qPCR) of blood samples, which can allow rapid and reliable detection of infection onset. While this can be effective, it is financially and logistically burdensome and not always feasible.

Many animal diseases manifest fever and weakness which may be difficult to detect by simple observation in early stages of infection. However, it can be detected through continuous-quantitative monitoring of animal behaviour through new technologies [8]. In this work, we aim to demonstrate that animals infected with ASF virus show a progressive deceleration in performing daily activities caused by muscle weakness from early stages of infection, which should translate into reduced overall motion, which quantitative automatic video processing should be able to detect [9]. This might allow detection of ASF onset in real time. Within the framework of the EU-funded Rapidia Field project (www.rapidia.eu), we continuously monitored eight pigs housed in a single indoor pen under Biosafety Level 3 (BSL-3) experimental conditions for 11 days before, and then 12 days after, experimental infection with ASF virus. Animal motion was quantified and classified using video processing and supervised learning, respectively, in order to detect significant early changes in behaviour between pre- and post-infection periods.

Lastest research and developments based on computer vision techniques have improved the current technology mainly designed for motion detection, object recognition and object tracking. With this work, we have implemented algorithms for motion capture, in which movement recorded by a camera is translated into digital models. It also incorporates a supervised learning method to classify motion-based animal behaviour. Finally, the system aims to assess the randomness of runs in order to detect a reduction in daily motion, such that a certain number of consecutive low motion scores may be attributable to ASF.

## Materials and methods

### Ethics statement

The experiment was carried out at the BSL-3 facilities of the VISAVET Centre of the Veterinary Faculty of the Universidad Complutense de Madrid (UCM). The *in vivo* experimental protocol was approved by the Committee of Animal Experimentation of the UCM (Regulation 2010/63/EU and Spanish Royal Decree 53/2013). The protocol included a detailed description of efforts to prevent unnecessary suffering of the animals, including humane endpoints and the use of anesthetics and euthanasic following Galindo-Cardiel et al. clinical evaluation guidelines [10]. Specifically, humane endpoints were reached when at least one parameter was scored as severe. Animals’ health and welfare was checked twice daily (morning and evening) by a clinical veterinarian. Pigs were also monitored 24h/day by videocameras that were checked regularly during the day.

### The experiment

The experiment lasted from 11 April 2015 (day 1) to 3 May 2015 (day 23), during which eight Landrace Large White healthy pigs from an authorized breeding centre (four months old and weighing approximately 40 kg) arrived together one day before and were enclosed together in one indoor pen maintained at constant temperature and humidity, with *ad libitum* access to food and water. They were continuously monitored using one camera in a fixed position. At least twice per day, animals were evaluated by veterinarians and technicians who monitored animal internal temperature, recorded clinical signs [11], kept the pen clean and controlled feed supplies. Once per day, blood and oral samples were analysed for ASF virus using a qPCR test [12], which allows the confirmatory diagnosis of ASF virus within hours of a sample.

All animals were accommodated in the pen and allowed to acclimate for one day before the experiment. On day 11, all animals were simultaneously inoculated with an attenuated strain of ASF virus from a tick captured in Kenya in 2005 (ASFV Ken05/Tk1). The first positive qPCR detection of the virus occurred on day 15 in six of eight animals. Subsequently, some animals showed mild clinical signs but first evident clinical signs associated with ASF started from day 18 in five of eight animals. After this day, all animals spent increasing amounts of time lying down until ultimately dying from the disease. The last animal died on day 23.

**Fig 1:**
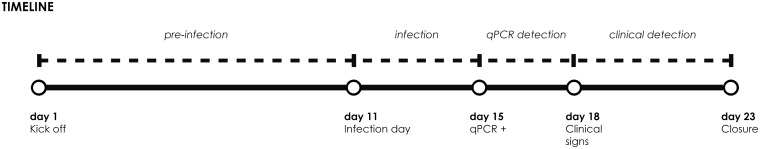
Timeline showing the four analytical phases of the experiment. The experiment began on day 1 (kick off), and animals were inoculated with ASF virus on day 11. On day 15, six of eight animals tested positive for the virus by quantitative PCR. On day 18, clinical signs of ASF became evident in five of eight animals. The experiment ended on day 23.

Based on this time course of infection, the experimental period was divided into 4 phases for analysis (Fig 1): (1) pre-infection (days 1-11), when animals were free of infection; (2) infection (days 12-15), after animals were inoculated with ASF virus but with no ASF virus detected; (3) qPCR detection (days 16-18), during which ASF virus was qPCR-detected in at least one animal; and (4) clinical detection (days 19-23), when clinical signs of ASF were evident in at least one animal, until the end of the experiment.

### Video filming

**Fig 2:**
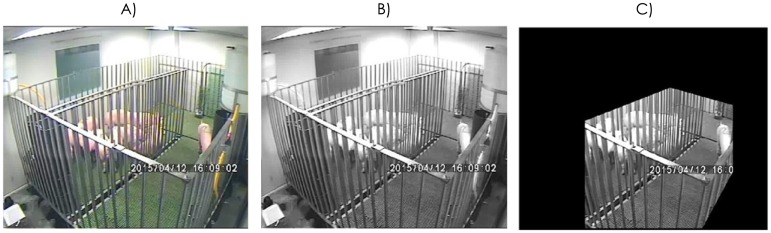
A) Original video frame (all RGB channels). B) The same frame in the red channel. C) Region of interest in the red-channel frame used for motion analysis.

A total of 541 hours of video footage were taken using a single camera equipped with night-vision capability at a fixed position in an upper corner of the pen (Fig 2A). Video footage was collected continuously throughout the 23-day experiment. The room was artificially illuminated from 7 a.m. to 9 p.m., while the camera recorded under night conditions from 9 p.m. to 7 a.m. The ‘U’-shape and the limited size of the pen as well as the tall metal enclosure did not allow individual tracking of the animals. Since the fixed camera filmed the same background throughout, and light intensity remained fairly constant within the day and night periods, it was easy to distinguish between animals in motion and static background, despite the standard definition (SD) resolution of the video which might make subsequent motion detection less sensitive.

Video was recorded at 6 frames per second (704 x 576 pixels) in RGB24 format, providing 24 bits in red, green and blue channels (RGB) for broad range of colour. We used the red channel for all analyses since it provided the best contrast for animal recognition (see Fig 2B). In addition, we analysed only a region of interest in which all animals were always observable (see Fig 2C).

### Motion capture

Animal movements were recorded and digitally processed through the Optical flow algorithm based on the Horn-Schunck methodology [13] implemented in Matlab. This algorithm estimates the speed and direction of moving objects between consecutive images based on the movement of brightness patterns [14]. This estimation process is described below.

Supposing that we know the value of a function *E*(*x*, *y*, *t*) determining the brightness of any point (*x*, *y*) in the (fixed) 2-dimensional domain *D* recorded in the video, at any time *t* ∈ [0, *T*_max_] during recording and considering a point following a trajectory (*x*(*t*), *y*(*t*)) in *D*, if this point maintains its brightness along the entire time interval, the value of *E*(*x*(*t*), *y*(*t*), *t*) remains constant for any *t* ∈ [0, *T*_max_]. Then, assuming that functions *E*, *x* and *y* are smooth,

$$\begin{array}{c} \frac{d}{dt}E\left(x\left(t\right),y\left(t\right),t\right)=0,\forall t\in \left[0,T_{\text{max}}\right], \end{array}$$ (1)

or, after applying the chain rule for differentiation,

$$\begin{array}{c} \frac{\partial E}{\partial x}\left(x\left(t\right),y\left(t\right),t\right)\frac{dx}{dt}\left(t\right)+\frac{\partial E}{\partial y}\left(x\left(t\right),y\left(t\right),t\right)\frac{dy}{dt}\left(t\right)+\frac{\partial E}{\partial t}\left(x\left(t\right),y\left(t\right),t\right)=0,\forall t\in \left[0,T_{\text{max}}\right]. \end{array}$$ (2)

In this equation, $\left(\frac{dx}{dt}\left(t\right),\frac{dy}{dt}\left(t\right)\right)$ represents the velocity of the point that at time *t* is in (*x*(*t*), *y*(*t*)). Since we cover the entire domain *D* with all possible trajectories, we deduce from Eq 2 that

$$\begin{array}{c} \frac{\partial E}{\partial x}\left(x,y,t\right)u\left(x,y,t\right)+\frac{\partial E}{\partial y}\left(x,y,t\right)v\left(x,y,t\right)+\frac{\partial E}{\partial t}\left(x,y,t\right)=0,\forall \left(x,y,t\right)\in D\times \left[0,T_{\text{max}}\right], \end{array}$$ (3)

where (*u*(*x*, *y*, *t*), *v*(*x*, *y*, *t*)) is the velocity of the point located at (*x*, *y*) ∈ *D* at time *t*. This can be expressed more compactly as

$$\begin{array}{c} E_{x}u+E_{y}v+E_{t}=0,\;\;\;\text{in}\;D\times \left[0,T_{\text{max}}\right]. \end{array}$$ (4)

To compute the velocity function (*u*, *v*), the Horn-Schunck method [13] minimises the error *ζ*(*u*, *v*) given by

$$\begin{array}{ccc} \zeta \left(u,v\right) & = & \int_{D}{\left(E_{x}u+E_{y}v+E_{t}\right)}^{2}\text{d}x\text{d}y+\eta^{2}\int_{D}\left(u_{x}^{2}+u_{y}^{2}+v_{x}^{2}+v_{y}^{2}\right)\text{d}x\text{d}y, \end{array}$$ (5)

where *η* is a weighting factor that scales global smoothness. Using the theory of calculus of variation, the velocity (*u*, *v*) minimising Eq 5 satisfies the Euler–Lagrange equations of *ζ*, given by

$$\begin{array}{c} \begin{array}{c} E_{x}^{2}u+E_{x}E_{y}v=\eta^{2}\Delta u-E_{x}E_{t},\\ E_{x}E_{y}u+E_{y}^{2}v=\eta^{2}\Delta v-E_{y}E_{t}, \end{array} \end{array}$$ (6)

where Δ*u* and Δ*v* are the Laplacians of *u* and *v*, defined as

$$\begin{array}{c} \Delta u=\frac{\partial^{2}u}{\partial x^{2}}+\frac{\partial^{2}u}{\partial y^{2}}\;\;\;\text{and}\;\;\;\Delta v=\frac{\partial^{2}v}{\partial x^{2}}+\frac{\partial^{2}v}{\partial y^{2}}. \end{array}$$ (7)

In reality, the entire video scene consists of $N_{\text{max}}\in N$ frames (or images) instead of continuous time *t* ∈ [0, *T*_max_], *E*(*x*, *y*, *t*) takes values in {0, 1, ⋯, 255} and *D* is divided into a matrix of 704 × 576 pixels. Therefore, we considered the discrete function *E*_*i*,*j*,*k*_ ∈ {0, 1, ⋯, 255} as the measured average brightness of the pixel at the intersection of the *i*th row and *j*th column in the *k*th frame. Hence, the minimum (*u*_*i*,*j*,*k*_, *v*_*i*,*j*,*k*_) of Eq 5 at an arbitrary pixel (*i*, *j*) in the discrete domain *D* of 704 × 576 pixels per frame *k* ∈ {0, 1, ⋯, *N*_max_ can be estimated iteratively as follows [13]:

$$\begin{array}{c} \begin{array}{c} u_{i,j,k}^{n+1}={\bar{u}}_{i,j,k}^{n}-E_{x}\frac{E_{x}{\bar{u}}_{i,j,k}^{n}+E_{y}{\bar{v}}_{i,j,k}^{n}+E_{t}}{\eta^{2}+E_{x}^{2}+E_{y}^{2}},\\ v_{i,j,k}^{n+1}={\bar{v}}_{i,j,k}^{n}-E_{y}\frac{E_{x}{\bar{u}}_{i,j,k}^{n}+E_{y}{\bar{v}}_{i,j,k}^{n}+E_{t}}{\eta^{2}+E_{x}^{2}+E_{y}^{2}}, \end{array} \end{array}$$ (8)

where $u_{i,j,k}^{0}=v_{i,j,k}^{0}=0$, *n* = {1, ⋯, *N*}, *N* = 25 is the maximum number of iterations, *η* = 10 considering a large relative motion between frames [15], and $\left({\bar{u}}_{i,j,k}^{n},{\bar{v}}_{i,j,k}^{n}\right)$ is the neighbourhood average of $\left(u_{i,j,k}^{n},v_{i,j,k}^{n}\right)$ computed using the convolution kernel $\left[1\;\sqrt{2}\;1;\;\sqrt{2}\;0\;\sqrt{2};1\;\sqrt{2}\;1\right]/4\left(1+\sqrt{2}\right)$ [16]. We computed (*E*_*x*_, *E*_*y*_) using the convolution kernel [−1 −2 −1;0 0 0;1 2 1] and its transposed form for each pixel in the first image and *E*_*t*_ between consecutive images using the [−1 1] kernel [15, 17].

Finally, we computed the global motion in frame *k* as

$$\begin{array}{c} m_{k}=\sum_{i=1}^{704}\sum_{j=1}^{576}\sqrt{u_{i,j,k}^{2}+v_{i,j,k}^{2}}. \end{array}$$ (9)

Eq 9 allows us to obtain a unique motion value for each frame *k* and to reduce motion analysis to time series analysis.

### Motion smoothing

We analysed the global motion, *m*_*k*_, in order to detect a significant reduction in animal motion following ASF infection. Nevertheless, two main facts had to be considered before the analysis. First, the video resolution caused a perturbation in the values of *m*_*k*_. Indeed, even when no motion was recorded, we observed that the background slightly changed between consecutive frames causing a problematic baseline noise in the time series. Second, as mentioned previously, the human factor altered the real values of global motion when the workers were in the region of interest of the screen. Thus, some intervals of time scored excessively high values of *m*_*k*_ only when the workers were in the pen.

**Fig 3:**
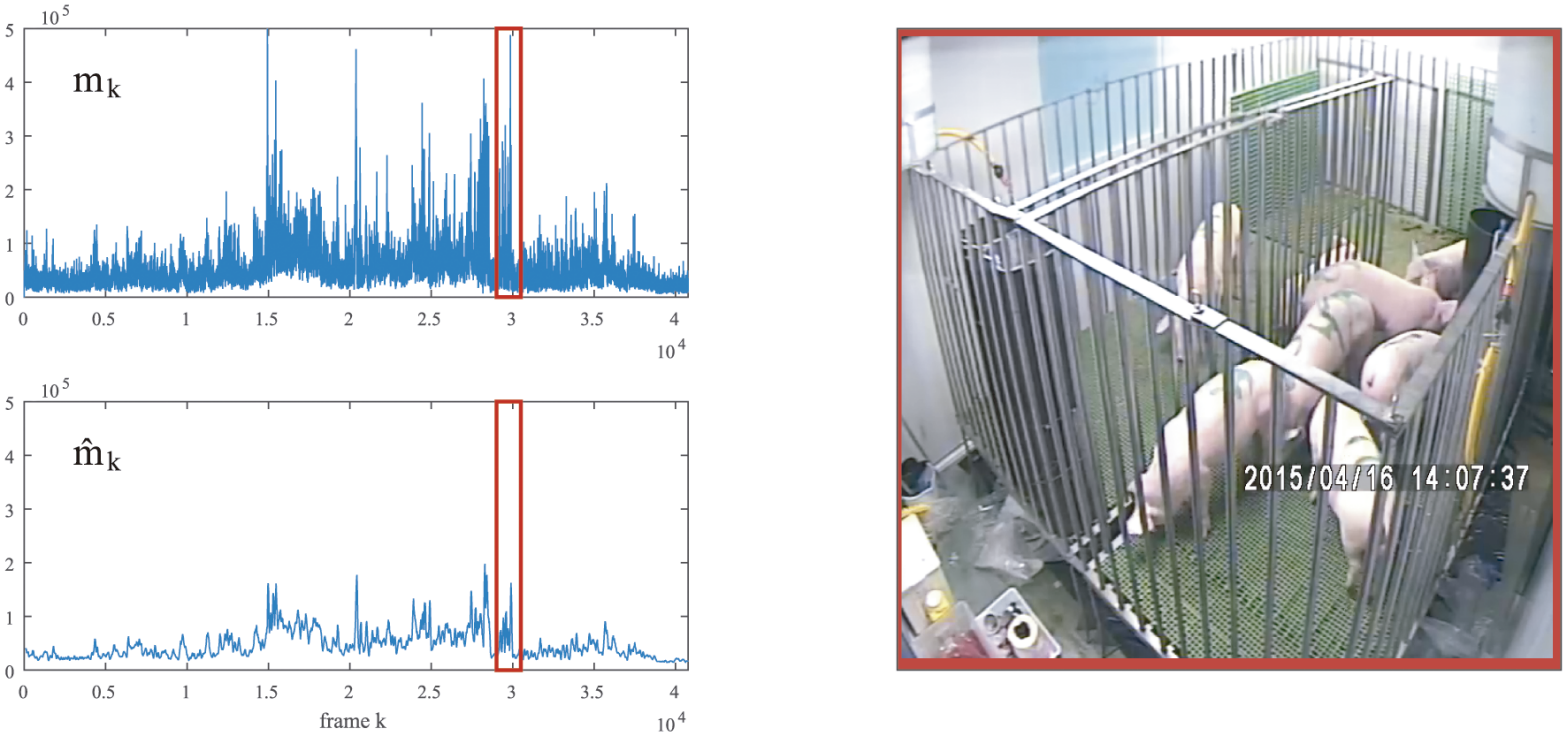
Global motion *m*_*k*_ and the corresponding moving average ${\hat{m}}_{k}$ for a randomly selected 2h of video footage. The moving average was performed over a window of *K* = 90 frames. The interval of time series boxed in red on the left correspond to the video image on the right, in which the animals were in motion.

To smooth the perturbation in *m*_*k*_, we considered a simple moving average filter by replacing each data point in the time series with the average of the previous K∈N data points (i.e. the previous *K* frames). In this work, we considered *K* = 90; that is, 15 seconds approximately (see Fig 3). Henceforth, all motion works were carried out throughout the new time series

$$\begin{array}{c} {\hat{m}}_{k}=\frac{1}{K}\sum_{i=0}^{K-1}m_{k-i},\forall k\in \left\{1,\cdots ,N_{\text{max}}\right\}. \end{array}$$ (10)

### Motion classification

Activity budgets relate to the use of an animal’s time including moving, standing or lying, feeding, drinking, social and aggressive behaviours [21]. In this work, three types of motion were proposed to classify animal activity during the experiment: (1) baseline, when all animals were lying or sleeping, such that motion was minimal or absent; (2) animal motion, when animals were walking, feeding or playing; and (3) human-related motion, when veterinary practices were carried out. Animal movements sometimes increased in the presence of humans, and also movements of the humans themselves contributed to overall motion when they entered the region of interest being recorded. Therefore we quantified the relative amounts of all three types of motion each day and sought to determine changes in animal motion unrelated to human interaction.

To classify motion as baseline, animal motion or human-related motion, we used short intervals of *S* = 15 seconds (corresponding to 6*S* = 90 frames) and we joined consecutive values of ${\hat{m}}_{k}$ into these intervals as follows

$$\begin{array}{c} M_{r}=[{\hat{m}}_{1+6S(r-1)},{\hat{m}}_{6Sr}],\forall r\in \left\{1,2,\ldots \right\}. \end{array}$$ (11)

We described each interval *M*_*r*_ using five statistical parameters [18]: minimum ($M_{r}^{\text{min}}$), first quartile ($M_{r}^{q1}$), median ($M_{r}^{\text{med}}$), third quartile ($M_{r}^{q3}$) and maximum ($M_{r}^{\text{max}}$).

Firstly, we analysed animal behaviour before experimental infection in order to quantify healthy levels of motion. To do this, we grouped the short series *M*_*r*_ corresponding to the pre-infection phase by using the *k*-means algorithm (*kmeans* command in Matlab performed with *squared Euclidean* distance) with the five statistical values ($M_{r}^{\text{min}}$, $M_{r}^{q1}$, $M_{r}^{\text{med}}$, $M_{r}^{q3}$, $M_{r}^{\text{max}}$), which assigned the input observations into *k* clusters through step-wise cluster centroid estimation based on optimisation of within- and between-cluster dispersion [19].

To set up the optimal number of clusters *k* used in the *k*-means algorithm, we used the gap criterion algorithm (*evalclusters* command in Matlab), which scored the so-called gap statistic associated to a proposed number of clusters based on the minimisation of within-cluster dispersion [20]. Consequently, the maximum value of the gap statistic is associated with the optimal number of clusters (assuming more than 3 and less than 50).

These *k* clusters should represent a different type of motion recorded during 15-seconds intervals, when the animals were not infected. For instance, intervals in which the animals showed a similar activity within 15 seconds should be grouped in the same cluster (i.e. where there is no high variation between $M_{r}^{\text{min}}$, $M_{r}^{q1}$, $M_{r}^{\text{med}}$, $M_{r}^{q3}$ and $M_{r}^{\text{max}}$). But depending on the number of animals in motion and the type of activity carried out, the mean value of motion may differ between intervals (i.e. $M_{r}^{\text{med}}$) and, consequently, such intervals may be grouped in different clusters. On the contrary, intervals in which the animals showed significant changes in the activity such as when they were walking and the human interaction accelerated the overall motion during an instant, should be grouped in clusters with a high variation between $M_{r}^{\text{min}}$, $M_{r}^{q1}$, $M_{r}^{\text{med}}$, $M_{r}^{q3}$ and $M_{r}^{\text{max}}$.

After *k*-means analysis, all *k* clusters (and the *M*_*r*_ records therein) were assigned to the three types of motion relevant for this work: baseline (group *G*_1_), animal motion (group *G*_2_) and (3) human-related motion (group *G*_3_).

Secondly, we classified the remaining *M*_*r*_ records to groups *G*_1_, *G*_2_ and *G*_3_ corresponding to post-infection periods (Fig 1). To do this, we built a support vector machine (SVM) pairwise classifier in Matlab.

To build an accurate SVM classifier, half of the *M*_*r*_ records in the pre-infection phase were randomly assigned to the so-called training dataset and the other half of records to the so-called testing dataset. To train the SVM model, we used the *svmtrain* command with the training dataset; then, we evaluated the accuracy of the model using the *svmclassify* command with the testing dataset. Once the accuracy of the model was verified, it was then used to classify the remaining *M*_*r*_ records of the experiment.

### Changes in animal motion as a result of infection

We expected that the daily activity budget (i.e. time spent lying/sleeping and feeding/walking) would change after infection with ASF virus. To measure this reliably, we used the Wald-Wolfowitz runs test (*runstest* command in Matlab) at significance levels of 90%, 95% and 99%, corresponding to respective p-values of less than 0.1, 0.05 or 0.01, which evaluated whether consecutive daily motion values were randomly distributed around the average value of the considered period. First, runs tests were performed during the pre-infection phase (days 1-11) to verify random distribution of motion values around the average value. Then, runs tests were repeated by expanding the window by one additional day until day 18, when clinical signs were detected. This allowed us to determine whether and when consecutive motion values significantly diverged from the average value, indicating ASF infection.

## Results

### Motion classification

**Fig 4:**
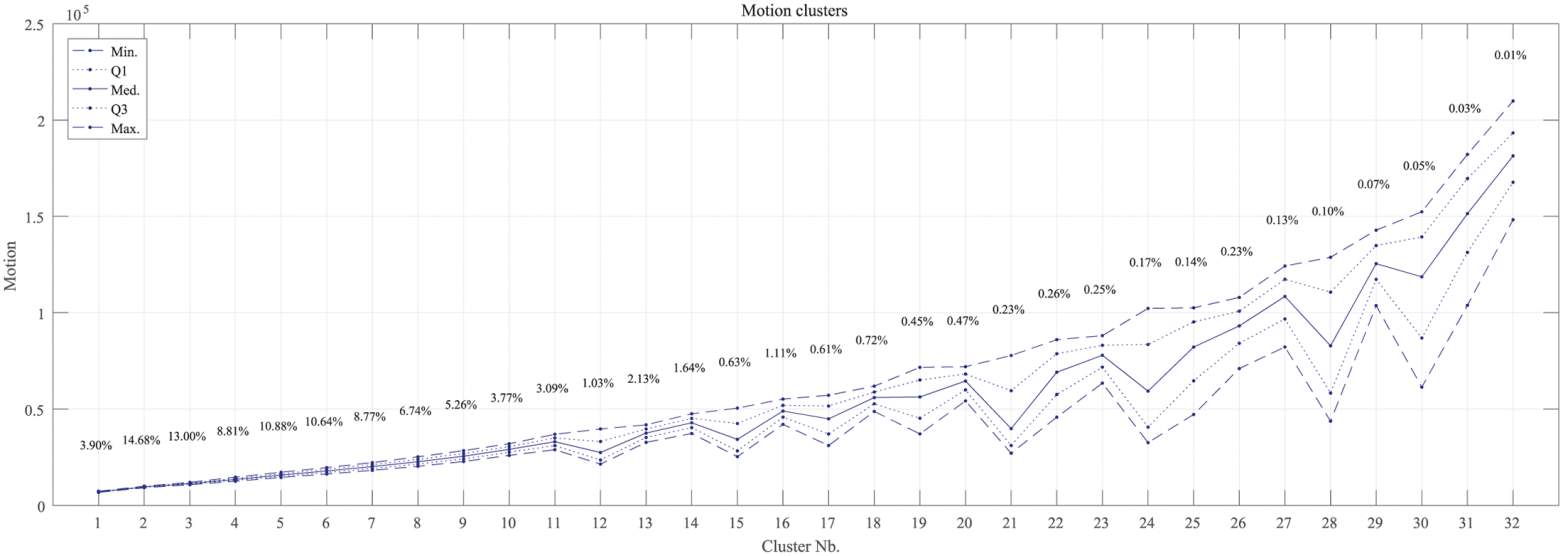
Minimum (Min.), first quartile (*Q*_1_), median (Med.), third quartile (*Q*_3_) and maximum (Max.) centroid values for the *k* = 32 clusters computed through *k*-means algorithm describing motion over 15-second intervals (90 frames) during the pre-infection period. Percentages refer to the total number of records in each cluster.

The gap statistic showed a maximum of 3.6955 for 32 clusters, with the next highest values being 3.6676 for 28 clusters and 3.6653 for 34 clusters. Therefore, we considered *k* = 32 clusters in the *k*-means algorithm. Fig 4 shows the centroids of these clusters and the percentage of *M*_*r*_ records classified in each one. After comparing many video sequences associated with *M*_*r*_ records across all clusters, we assigned cluster 1-7 to baseline (*G*_1_), clusters 8-19 to animal motion (*G*_2_), and clusters 20-32 to human-related motion (*G*_3_). As expected, the lowest motion values corresponded to the baseline, which occurred during 70.06% of the pre-infection period, i.e. around 70% of their time inactive [21]. Intermediate motion values corresponded to animal motion, which occurred during 27.67% of the pre-infection period. Finally, human-related motion was greatest occurring during 2.27% of the time.

The SVM classifier showed accuracy of 95.66–98.87% at classifying records to groups *G*_1_, *G*_2_ and *G*_3_ (Table 1). Conversely, only 1.64% of records in *G*_1_ were incorrectly assigned to *G*_2_, 1.13% of records in *G*_3_ were incorrectly assigned to *G*_2_ and records in *G*_2_ were incorrectly assigned to *G*_1_ (1.14%) or *G*_3_ (3.20%).

**Table 1:** Percentages of records in the testing dataset correctly and incorrectly assigned to groups *G*_1_, *G*_2_ and *G*_3_ by the pairwise SVM- classifier.

group	classified in group		
	*G*_1_	*G*_2_	*G*_3_
*G*_1_	98.36%	1.64%	0.00%
*G*_2_	1.14%	95.66%	3.20%
*G*_3_	0.00%	1.13%	98.87%

### Changes in animal motion as a result of infection

Table 2 shows the proportions of time spent each day in baseline, animal motion and human-related motion computed with the entire data set. After the virus inoculation, lying/sleeping time increased substantially and feeding/walking the time where during which animals were in motion decreased substantially across the following post-infection phases. In fact, in the first four days the animal motion decreased 8.51 points and, before the detection through clinical signs, 9.97 points respect to the pre-infection phase.

**Table 2:** Percentage of time spent each day in baseline, animal motion and human-related motion for each of the four experimental phases defined in Fig 1.

Type of motion	Pre-infection	Infection	PCR detection	Clinical detection
Baseline	70.06%	79.33%	80.72%	88.55%
Animal	27.67%	19.16%	17.70%	9.60%
Human-related	2.27%	1.51%	1.58%	1.85%

**Fig 5:**
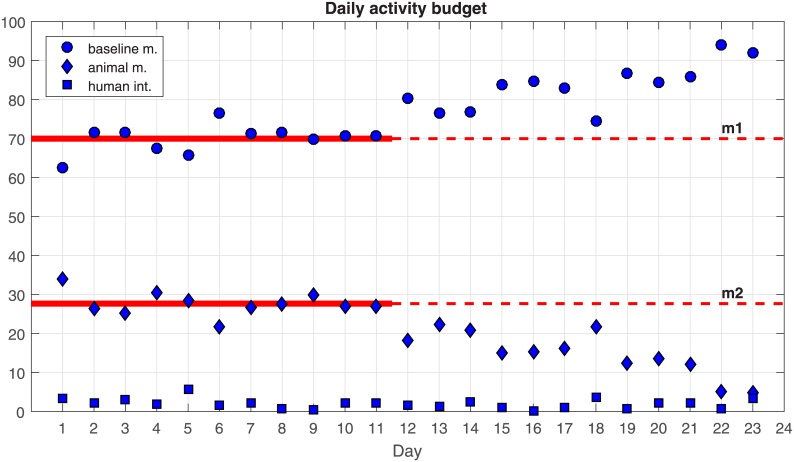
Percentage of time each day involving baseline, animal motion or human-related motion. Mean values over the first 11 days are shown as red lines for baseline (*m*_1_) and animal motion (*m*_2_).

Comparison of the percentages of records *M*_*r*_ classified by the SVM approach into groups *G*_1_, *G*_2_ and *G*_3_ for each day of the experiment (Fig 5) shows that human-related motion occurred during only a fraction of the time, but it made a disproportionate contribution to the amount of motion generated, covering the clusters with the highest motion values, ${\hat{m}}_{k}$. Therefore, such motion was excluded from the analysis. We executed the SVM-classifier for the remaining days of the experiment. Fig 5 shows the percentage of records, *M*_*r*_, classified in groups *G*_1_, *G*_2_ and *G*_3_ per day of the experiment; that is, the time classified as baseline motion, animal in motion and human interaction per day. Throughout the experiment, the human interaction represented a small percentage of the records.

#### Pre-infection phase (days 1-11)

Aside from day 1, when animals were slightly hyperactive because of their new environment, they spent fairly constant proportions of each day lying/sleeping and feeding/walking. These proportions varied randomly around the respective mean values of *m*_1_ and *m*_2_.

#### Infection phase (days 12-15)

Following experimental infection with ASF virus, the proportion of time spent each day in baseline tended to increase, while the proportion of time spent in animal motion tended to decrease. Thus, the percentage of time each day in baseline was higher than on any other previous day of the experiment, while the percentage of time in animal motion was lower than on any other previous day, pointing to a probable effect of ASF.

#### PCR detection phase (days 16-18)

The trends observed during the infection phase continued during the PCR detection phase. Percentages of baseline and animal motion remained quite high and low, respectively.

#### Clinical detection phase (days 19-23)

The trends observed during the infection phase and PCR detection phase worsened during the clinical detection phase, with the percentage of baseline increasing and percentage of animal motion decreasing. This worsening should be interpreted with caution because the animals began to die rapidly from day 18 onwards. In fact, two animals died at day 19 and six at day 23. Therefore the data collected during this phase is unlikely to be useful for assessing the ability of our approach to detect ASF early.

#### Runs test

Runs tests were carried out for progressively longer time windows during the 23-day recording period in order to examine on what day the changes in baseline and animal motion deviated significantly from pre-infection values (Table 3). These runs tests focused on days 11-18, the period critical for early ASF detection (Fig 5). Runs tests for the period from days 1 to 11 suggested that, as expected, percentages of baseline and animal motion varied randomly around their mean values. Runs tests for longer periods indicated that although the percentage of baseline increased after inoculation and the percentage of animal motion, these changes did not deviate significantly from pre-infection values until day 15, when the differences became significant at the 90% level. The differences were significant at the 95% level on day 16 and at the 99% level on day 18.

**Table 3:** The |*Z*| statistic and 2-tailed significance (Sig.) for runs tests of percentages of daily time spent in baseline or animal motion (Fig 5). Runs tests were performed on progressively longer intervals of the experimental period, from days 1-11 to days 1-18.

Runs	Day interval							
Baseline	[1, 11]	[1, 12]	[1, 13]	[1, 14]	[1, 15]	[1, 16]	[1, 17]	[1, 18]
|*Z*| Sig. (2-tailed)	0.28 0.77	0.11 0.90	-0.02 0.97	-0.14 0.88	-0.10 0.91	-0.19 0.84	-2.45 0.01**	-2.65 0.01***
Animal motion	[1, 11]	[1, 12]	[1, 13]	[1, 14]	[1, 15]	[1, 16]	[1, 17]	[1, 18]
|*Z*| Sig. (2-tailed)	-0.61 0.54	-0.20 0.83	-0.56 0.57	-0.83 0.10	-2.13 0.03**	-2.33 0.02**	-2.49 0.01**	-2.65 0.01***

*, ** and ***: significance at 90%, 95% and 99%, respectively.

## Discussion

Video processing of livestock offers the possibility of non-intrusive animal monitoring in real time. Here we aimed to examine whether such monitoring could reliably detect decreases in pig motion in the early stages of ASF infection. Our results in this pilot study showed that, indeed, we detected a significant decrease in motion at just four days after experimental infection with the ASF virus. This was the same day that the virus was detected in blood using qPCR, and a full three days before clinical signs of ASF were observed.

The proposed method comprises motion estimation, clustering/classification of motion patterns and recognition of these patterns via supervised learning. “Optical flow” is a widely used algorithm to measure the apparent velocities of objects in video streams that allowed us to quantify motion between consecutive frames throughout the entire experiment. We divided the total recording period into intervals of a few seconds and classified motion in each interval into (1) baseline (little or no motion), (2) animal motion and (3) human-related motion. An SVM pairwise classifier was used to train the model to recognise the three types of motion based on a randomly selected 50% of the pre-infection dataset, and this ability was then assessed using the other half of the same dataset. After establishing an accuracy >95%, we applied the classifier to the data collection during the post-infection phases of the experiment. This allowed us to detect an increase in the proportion of baseline activity and concomitant decreases in animal motion. To assess the possibility that these results might be due only to chance, we performed runs tests on data over progressively longer time windows. This analysis indicated that the changes in motion attributable to ASF became significant at four days after infection (95% level).

In our study, a slight perturbation in the global motion was recorded, even when no animals were in the pen (before the start of the experiment) or when the animals did not move, probably reflecting the SD resolution of the camera. Our efforts to treat these perturbed time series as retrogressive models (ARMA/ARIMA) were unsatisfactory, so we treated them as normally distributed and as contributing a constant amount of daily motion to the observed total. It is possible that alternative approaches to noise reduction may allow more refined classification and recognition of motion types, such as differentiation between feeding and walking. In an ideal case, such approaches should be adaptable to a range of farm and animal types through machine learning algorithms, as used here. On the other hand, the runs tests used here were enough to detect changes in 11 days but, considering field conditions, i.e. longer periods in farms, it could be more effective the monitoring through quality control techniques.

Likely important to the success of our approach is filtering out animal motion largely or entirely induced by veterinarians and technicians visiting the pen, as well as movement of these workers mistakenly recorded as animal motion during the experiment. While in practice only a fraction of daily motion in our study was human-related, this motion occasionally produced false spikes and so needed to be removed. Future work should examine the possibility of extending Optical flow through the implementation of animal tracking and recognition algorithms [22, 23] in order to eradicate definitely human-related motion and to enhance the individual animal monitoring. Therefore, our results with an SD resolution camera suggest that future work should focus on high definition (HD) cameras, which can automatically adjust contrast and brightness and so which may further reduce background noise and blurring.

The technology developed here has been tested in an experimental environment with 8 animals during a short period. Studies related with ASF infected animals do not usually consume long periods nor use high herd sizes. But in field conditions, different issues may vary the motion scores such as the increment of the number of animals monitored, the apparition of any other animal interaction, climatic factors that may alter the light intensity, etc. All these factors should be considered in future works to verify that this system is useful to detect disease outbreaks in field conditions.

Our results illustrate the promise of video processing for early detection of ASF. Many other livestock diseases might present slowdown in motion caused by fever in animals, such as foot-and-mouth disease, classical swine fever, Rift Valley fever, African horse sickness or bluetongue. The possibility to extend the technology presented here to detect rapidly other diseases might improve the current surveillance protocols substantially. In fact, we showed that four days after inoculation, the system launched an alert of ASF-suspicion that, under real farm conditions, probably could be interpreted for the veterinary services to take blood test and confirm an outbreak three days before the first clinical signs.

## Supporting information

S1 File Tables and scripts. Files containing raw data, main processed data and main scripts in Matlab.
(ZIP)

## References

[1] Fernández-Carrión E, Ivorra B, Martínez-López B, Ramos AM, Sánchez-Vizcaíno JM. Implementation and validation of an economic module in the Be-FAST model to predict costs generated by livestock disease epidemics: Application to classical swine fever epidemics in Spain. Preventive veterinary medicine 2016;126:66–73. 10.1016/j.prevetmed.2016.01.015 26875754

[2] Gibbens JC, Sharpe CE, Wilesmith JW, Mansley LM, Michalopoulou E, Ryan JB, Hudson M. Descriptive epidemiology of the 2001 foot-and-mouth disease epidemic in Great Britain: the first five months. The Veterinary Record 2001;149(24):729–743. 11808655

[3] McLaws M, Ribble C, Martin W, Wilesmith J. Factors associated with the early detection of foot-and-mouth disease during the 2001 epidemic in the United Kingdom. The Canadian Veterinary Journal 2009;50(1):53 19337614 PMC2603653

[4] Stegeman A, Elbers A, De Smit H, Moser H, Smak J, Pluimers F. The 1997–1998 epidemic of classical swine fever in the Netherlands. Veterinary Microbiology 2000;73(2):183–196. 10.1016/S0378-1135(00)00144-9 10785327

[5] Elbers ARW, Stegeman A, Moser H, Ekker HM, Smak JA, Pluimers FH. The classical swine fever epidemic 1997–1998 in the Netherlands: descriptive epidemiology. Preventive veterinary medicine 1999;42(3):157–184. 10.1016/S0167-5877(99)00074-4 10619154

[6] Sánchez-Vizcaíno JM, Mur L, Martínez-López B. African swine fever (ASF): five years around Europe. Veterinary microbiology 2013;165(1):45–50. 23265248 10.1016/j.vetmic.2012.11.030

[7] World Organisation for Animal Health Guide to terrestrial animal health surveillance 2014.

[8] Matthews SG, Miller AL, Clapp J, Plötz T, Kyriazakis I. Early detection of health and welfare compromises through automated detection of behavioural changes in pigs. The Veterinary Journal 2016;217:43–51. 10.1016/j.tvjl.2016.09.005 27810210 PMC5110645

[9] Martínez-Avilés M, Fernández-Carrión E, López García-Baones JM, Sánchez-Vizcaíno JM. Early detection of infection in pigs through an online monitoring system. Transboundary and emerging diseases 2017;64(2):364–373. 10.1111/tbed.12372 25955521

[10] Galindo-Cardiel I, Ballester MA, Solanes D, Nofrarías M, López-Soria S, Argilaguet JM, Lacasta A, Accensi F, Rodríguez F, and Segalés J. Standardization of pathological investigations in the framework of experimental ASFV infections. Virus research 2013;173(1):180–190. 10.1016/j.virusres.2012.12.018 23313935

[11] Penrith ML, Nyakahuma D. Recognizing African swine fever: a field manual. Food & Agriculture Org 2000;9.

[12] King DP, Reid SM, Hutchings GH, Grierson SS, Wilkinson PJ, Dixon LK, Bastos ADS, Drew TW. Development of a TaqMan^®^ PCR assay with internal amplification control for the detection of African swine fever virus. Journal of virological methods 2003;107(1):53–61. 10.1016/S0166-0934(02)00189-1 12445938

[13] Horn BKP, Schunck BG. Determining optical flow. Artificial intelligence 1981;17(1-3):185–203. 10.1016/0004-3702(81)90024-2

[14] Gibson JJ. The perception of the visual world. Houghton Mifflin 1950.

[15] Khobragade AS, Kulat KD, Dethe CG. Motion analysis in video using optical flow techniques. International Journal of Information Technology and Knowledge Management 2012;5(1):9–12.

[16] Karlsson SM, Bigun J. Lip-motion events analysis and lip segmentation using optical flow. IEEE Computer Society Conference on Computer Vision and Pattern Recognition Workshops 2012;138–145.

[17] Farid H, Simoncelli EP. Optimally rotation-equivariant directional derivative kernels. International Conference on Computer Analysis of Images and Patterns 1997;207–214.

[18] Siuli S, Li Y, Wen P. Clustering technique-based least square support vector machine for EEG signal classification. Computer methods and programs in biomedicine 2011;104(3):358–372. 10.1016/j.cmpb.2010.11.014 21168234

[19] Lloyd S. Least squares quantization in PCM. IEEE transactions on information theory 1982;28(2):129–137. 10.1109/TIT.1982.1056489

[20] Tibshirani R, Walther G, Hastie T. Estimating the number of clusters in a data set via the gap statistic. Journal of the Royal Statistical Society: Series B (Statistical Methodology) 2001;63(2):411–423. 10.1111/1467-9868.00293

[21] Maselyne J, Saeys W, De Ketelaere B, Mertens K, Vangeyte J, Hessel EF, Millet S, Van Nuffel A. Validation of a High Frequency Radio Frequency Identification (HF RFID) system for registering feeding patterns of growing-finishing pigs. Computers and Electronics in Agriculture 2014;102:10–18. 10.1016/j.compag.2013.12.015

[22] Stauffer C, Grimson W, Eric L. Adaptive background mixture models for real-time tracking. Computer Vision and Pattern Recognition 1999;2:246–252.

[23] Viola P, Jones M. Rapid object detection using a boosted cascade of simple features. Computer Vision and Pattern Recognition 2001;1:1–511.

